# P-895. CARE PATH: Paving a Way to Better Antibiotic Stewardship

**DOI:** 10.1093/ofid/ofaf695.1103

**Published:** 2026-01-11

**Authors:** Michael J Bozzella, Joana Dimo, Rachel Schaefer, Lauren Biehle, Matthew J Weber, Matthew Miller, Kira Frappa, Leigh Anne Bakel, Christopher A Czaja, Sarah K Parker

**Affiliations:** Children's Hospital Colorado, Aurora, CO; University of Colorado/Children's Hospital Colorado, Denver, Colorado; Colorado Department of Public Health and Environment, Denver, Colorado; Colorado Department of Public Health and Environment, Denver, Colorado; University of Colorado/Children's Hospital Colorado, Denver, Colorado; Children's Hospital Colorado, Aurora, CO; Children's Hospital Colorado, Aurora, CO; University of Colorado/Children's Hospital Colorado, Denver, Colorado; Colorado Department of Public Health and Environment, Denver, Colorado; University of Colorado/Children's Hospital Colorado, Denver, Colorado

## Abstract

**Background:**

CDC Core Elements of Hospital Antibiotic Stewardship (AS) and national accreditation bodies prioritize the implementation of facility-specific treatment recommendations and monitoring of adherence to those recommendations. These activities are a challenge for resource-limited hospitals, which we sought to overcome with the Colorado Antimicrobial Resources, Education, and Pathway Adherence Tools for Hospitals (CARE PATH) initiative. Here we report interim findings.

**Table 1:**
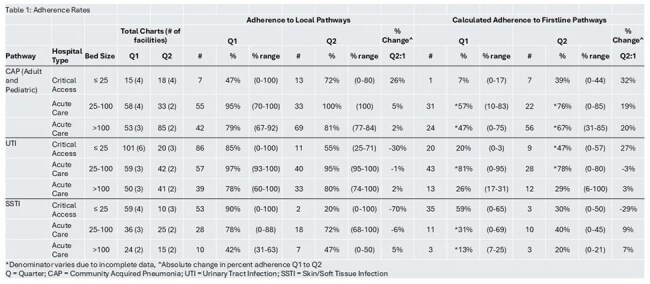
Adherence Rates Breakdown of adherence rates to clinical pathways by hospital type

**Figure 1:**
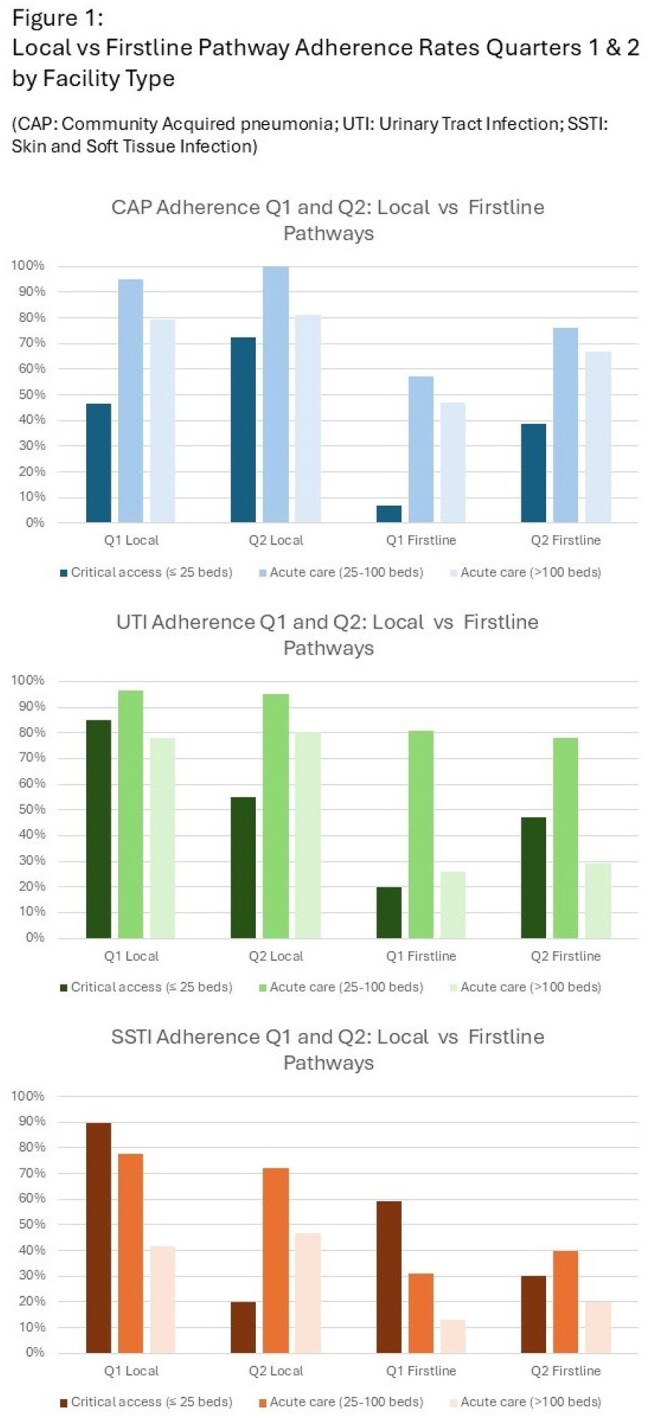
Local vs Firstline Pathway Adherence Rates Quarters 1 and 2 by Facility Type Graphic representation of pathway adherence rates by disease state, pathway type, and facility type

**Methods:**

Children’s Hospital Colorado, in partnership with Denver Health, created a free mobile application using Firstline (Firstline.org) to share evidence-based clinical pathways across Colorado, which could be adapted for use as facility-specific treatment recommendations.

From July 2024-March 2025 hospitals voluntarily submitted antibiotic use data for cases of pediatric or adult community acquired pneumonia (CAP), urinary tract infection (UTI), and/or skin/soft tissue infection (SSTI) via a web-based REDCap survey. AS experts assessed prescribing compared to local clinical pathways (LCPs) and Firstline. LCP adherence was based on self-report of antibiotic selection. Firstline adherence was met if 3 criteria were consistent with app recommendations: antibiotic selection, treatment duration, and clinical indication. Facilities received quarterly reports with feedback and recommendations for AS. To supplement the reports, our team hosted a series of interactive webinars and 1-on-1 mentorship sessions.

**Results:**

26 facilities registered for CARE PATH, 11 critical access (42%) and 15 acute care (58%). 22 (85%) reported having a local adult or pediatric CAP pathway, 21 (81%) a UTI, and 21 (81%) a SSTI LCP. 12 (46%) did not monitor adherence prior to CARE PATH. 13 (50%) facilities reported data on at least one condition in quarter 1, and 8 (30%) for quarter 2. Adherence rates to LCPs and Firstline are presented in Table 1 and Figure 1.

**Conclusion:**

CARE PATH has helped 13 Colorado hospitals meet priority core elements. Adherence rates varied, with SSTI the lowest. CAP adherence increased Q1 to Q2, though UTI and SSTI changes were variable. These findings represent a novel hospital-state health collaboration and inform state-wide antimicrobial stewardship efforts.

**Disclosures:**

Leigh Anne Bakel, MD, Colorado Department of Public Health and Environment: Grant/Research Support|Pfizer: Grant/Research Support Sarah K. Parker, MD, Colorado Department of Health and Environment: Advisor/Consultant|Infectious Diseases Society of America: Reimbursement for Program Committee|Pfizer Global Bridges: Grant/Research Support|Society for Healthcare Epidemiology of America: Honoraria|Society of Infectious Diseases Pharmacists: Honoraria

